# Rapid evolution allows coexistence of highly divergent lineages within the same niche

**DOI:** 10.1111/ele.14061

**Published:** 2022-06-27

**Authors:** Ben A. Ward, Sinead Collins

**Affiliations:** ^1^ School of Ocean and Earth Science University of Southampton Southampton UK; ^2^ Institute of Evolutionary Biology, School of Biological Sciences University of Edinburgh Edinburgh UK

**Keywords:** biodiversity, coexistence, convergent evolution, functional redundancy, microbial, neutral

## Abstract

Marine microbial communities are extremely complex and diverse. The number of locally coexisting species often vastly exceeds the number of identifiable niches, and taxonomic composition often appears decoupled from local environmental conditions. This is contrary to the view that environmental conditions should select for a few locally well‐adapted species. Here we use an individual‐based eco‐evolutionary model to show that virtually unlimited taxonomic diversity can be supported in highly evolving assemblages, even in the absence of niche separation. With a steady stream of heritable changes to phenotype, competitive exclusion may be weakened, allowing sustained coexistence of nearly neutral phenotypes with highly divergent lineages. This behaviour is robust even to abrupt environmental perturbations that might be expected to cause strong selection pressure and an associated loss of diversity. We, therefore, suggest that rapid evolution and individual‐level variability are key drivers of species coexistence and maintenance of microbial biodiversity.

## Graphical abstract

Here we use an individual‐based eco‐evolutionary model in which taxonomic diversity emerges as a consequence of evolutionary history. Using this model we are able to show that virtually unlimited genetic divergence can be emerge through convergent evolution in highly abundant and rapidly evolving assemblages, even in the absence of niche separation.

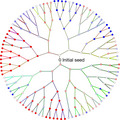

## INTRODUCTION

Marine microbial communities are a fundamental driver of global biogeochemical cycles. Photosynthetic plankton form the energetic foundation of virtually all pelagic ecosystems, while cycling among broader networks of individuals plays a key role in the regulation of Earth's climate (Guidi et al., 2016). While individual metabolic processes and functional traits are often well correlated with environmental conditions (Cohen et al., 2021; Marañón et al., 2012; Thomas et al., 2012; Ustick et al., 2021), our ability to predict when and where individual taxa become important is complicated by an extremely high degree of taxonomic diversity. Indeed, among the approximately 10^28^ microbial cells living in the ocean (Flombaum et al., 2013), recent bioinformatic surveys have identified the existence of up to 150,000 genera of marine eukaryotes in the photic layer alone (de Vargas et al., 2015).

In addition to this raw taxonomic diversity, globally important metabolisms and functional traits often appear broadly distributed across the tree of life, and in any given environment may be performed equally well by a large number of individual taxa. There is thus a high degree of functional redundancy in marine ecosystems (Louca et al., 2016), with the selection of traits and function occurring irrespective of taxonomic classification. For example, global metagenomic analysis points to high taxonomic dissimilarity among functionally very similar communities (Louca et al., 2018; Sunagawa et al., 2015). Likewise, single‐cell genomic analyses have shown extremely high levels of genetic divergence among coexisting cells from the same taxonomic group (Kashtan et al., 2014; Rynearson & Armbrust, 2000).

This pattern of functional redundancy brings a new perspective to a longstanding question in marine microbial ecology, namely ‘*how it is possible for a number of species to coexist in a relatively isotropic or unstructured environment all competing for the same sorts of materials*’? (Hutchinson, 1961). As initially suggested by Hutchinson himself, many valid solutions to this ‘paradox’ exist (Record et al., 2013). Species compete for (and are limited by) a broad range of chemical and biological factors that enable coexistence (Tilman, 1977). It is also clear that even a well‐mixed ocean is neither isotropic nor unstructured (d'Ovidio et al., 2010). Spatial partitioning can thus occur at many different scales and ecological equilibrium is often prevented by external perturbations (Litchman et al., 2009) and internal dynamics (Huisman et al., 1999) such that competitive exclusion can be indefinitely postponed.

The mechanisms above work by partitioning coexisting species into different niches or by separating them in time or physical space, but do not address the potential for more than one species to coexist within a single niche. An alternative perspective, provided by the neutral theory of biodiversity (Hubbell, 2001), suggests that an unlimited degree of diversity can be maintained within the same niche if species have effectively identical fitness in their shared environment.

While the neutral theory can explain some observed patterns of diversity (Hubbell, 2001), it is often criticized on the grounds that even tiny differences in fitness must eventually lead to competitive exclusion (in the absence of other mechanisms; Hardin, 1960; Loreau, 2004). This is argued to be particularly true in microbial populations, for which huge population sizes tend to diminish the importance of stochastic effects that might delay exclusion (Louca et al., 2018). All other things being equal, even a relatively small increase in fitness is expected to rapidly fix within the population, in a selective sweep (Hermisson & Pennings, 2017; Louca et al., 2018).

While these ecological considerations suggest that neutrality is an unlikely outcome in microbial communities, the degree to which species can coexist is also known to be affected by evolution (Kremer & Klausmeier, 2017). Laboratory cultures may display a high level of phenotypic convergence among traits that are strongly correlated with fitness (Blount et al., 2018), suggesting differences in many trait values and their associated fitness may be minimized through time. On one hand, convergent evolution can maintain diversity by eliminating the fitness differences that lead to exclusion (Hubbell, 2006; Scheffer & van Nes, 2006). On the other, the same processes can eliminate complementary differences in phenotype that support coexistence, thus driving a steady decline in biodiversity (Sauterey et al., 2014; Shoresh et al., 2008). Among these modelling studies, a common feature is that the evolving community is represented as discrete populations differentiated by ecophysiological traits. This precludes the examination of potentially important processes of birth, death and mutation occurring at the individual level, or of the substantial variation known to underlie a given set of trait values. These individual‐level processes require consideration. For example, individual‐based models (IBMs) have shown that phenotypic noise among individuals in large populations may be sufficient to add variation to the outcomes of local competitions, allowing extended coexistence of highly similar populations (or even populations of equal average fitness) within the same niche (Menden‐Deuer et al., 2021). This suggests that competitive exclusion may proceed much more slowly given realistic levels of noise between genotype and phenotype when populations have the same or very similar average fitnesses (although this does not explain why small differences in average fitness would not eventually lead to exclusion).

In this article, we address questions of functional and taxonomic diversity using an ecological and evolutionary (eco‐evo) model that makes no prior assumptions regarding the differentiation of populations, species or ecotypes. Instead, the community is resolved at the individual level, with species and populations treated as emergent properties based on genetic rather than phenotypic distance. To achieve this, we take an established eco‐evolutionary model (Beckmann et al., 2019) and add a neutral genomic component that allows us to track descent and diversity under a range of scenarios. With simulations based on realistic ecophysiological parameters, we show that virtually unlimited diversity is a natural consequence of highly abundant evolving populations, with rapid trait evolution allowing lineages to avoid competitive exclusion despite even sharp changes in environmental conditions.

### An individual‐based model of microbial evolution

**FIGURE 1:**
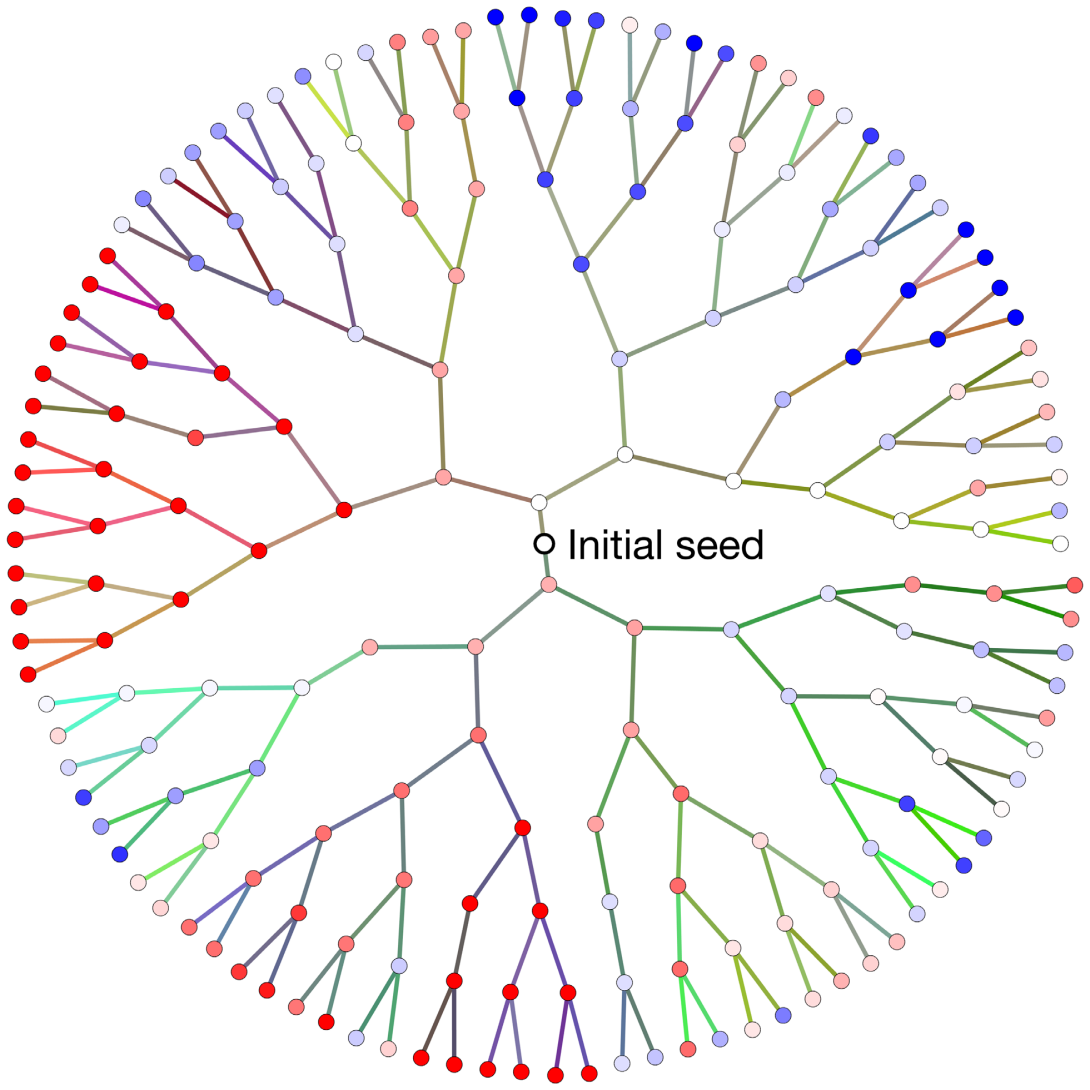
Genealogy in the IBM after 7 days of growth at a constant environmental temperature. Terminal nodes at the perimeter represent live cells that have descended from the initial seed at the centre. Each non‐terminal node represents a cell division, with branch lengths linearly proportional to the time between divisions. Nodes are coloured according to the thermal optimum of each dividing cell (red prefers warmer and blue prefers colder). Branch colours correspond to value of the neutral rgb gene. Note that branch colours change gradually along branches, such that related individuals have similar colours. Extinct lineages are not shown.

The eco‐evolutionary model provides a very simple representation of a closed marine microbial ecosystem, with state variables for nutrients, individual phytoplankton cells and organic detritus (Beckmann et al., 2019). The phytoplankton community is represented as a collection of individual cells that take up nutrients and increase in size as a function of their environmental conditions and ecophysiological traits. Cells divide into two daughter cells once they have doubled in biomass relative to a predefined threshold. Cells die through a stochastic process, producing organic detritus that is remineralised to inorganic nutrient at a fixed linear rate. Individual cells differ only in terms of their optimal temperature for growth, which is passed from generation to generation with some error, allowing for evolution by selection (Figure 1). Here, heritable variation is modelled as a random walk in a one‐dimensional trait space, which represents the organisms' thermal optima. Heritable changes in trait values may be attributable to any combination of genetic and epigenetic mutations, as well as transgenerational plasticity that can affect the trait in question. These changes need not correspond directly to genetic point mutation rates, but rather to the per‐generation rate of trait value change, which can be affected by all or some of these processes. Hereafter, we refer to heritable trait changes generically as ‘mutation’, regardless of the molecular cause of the change. A more detailed description of the individual‐based model can be found in Appendix A and Beckmann et al. (2019).

In addition to the model components laid out by Beckmann et al. (2019), each simulated individual is assigned two heritable but ecologically neutral characteristics: a binary string that undergoes a single random bit flip at each generation, and a ‘colour trait’ encoded as a three‐element vector (red, green and blue) that also varies randomly from generation to generation (see Methods). The binary genome can be thought of as representing a two‐base equivalent to a non‐coding RNA or DNA sequence. Given that (a) genomes are identical at the point of division, (b) changes in the genomes are not under selection and (c) genomes acquire mutations at a fixed rate, the binary genome can be used as a molecular clock. Changes through time accrue according to a 2‐base version of the Jukes and Cantor (1969) model of base substitutions (Appendix A). The colour trait is included primarily for visualization, with closely related individuals appearing with similar colours (Figure 1).

### Phenotypic and genotypic diversity within a single niche

Beckmann et al. (2019) initially ran their model with a total nutrient load of 5 μM N and a constant environmental temperature of 15°C. The model converged to a steady‐state with individuals occupying a Gaussian distribution of thermal optima (15 ± 0.855°C) centred on the environmental temperature. We repeated this experiment, running the model for 1000 years and obtaining an identical trait distribution.

**FIGURE 2:**
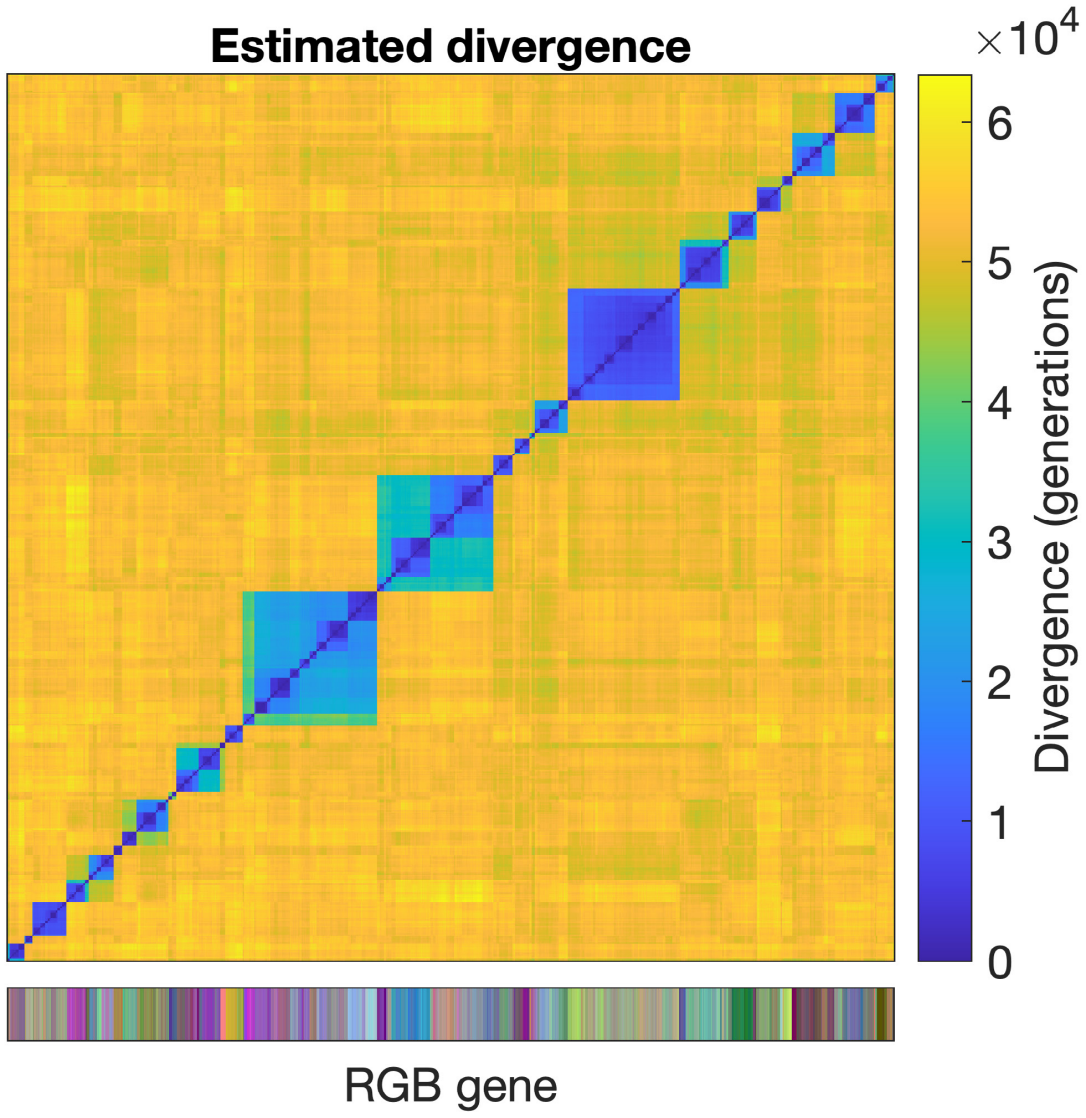
Estimated divergence matrix for 1000 cells sampled at the end of a 1000‐year simulation, as derived from the binary genome. The estimated number of generations since the most‐recent common ancestor is shown according to the right‐hand colour scale. The lower colour scale shows each individual's neutral colour trait.

We use the new bioinformatic components of the model to explore the mechanisms by which this trait distribution arises. Using the neutral binary genome to estimate the genealogy of the population, Figure 2 shows the estimated pairwise distance matrix for 1000 individuals sampled at the end of the 1000‐year simulation. Although the simulation only includes a single thermal niche, we see multiple distinct genotypic clusters coexisting within that niche, each with many tens of thousands of generations worth of genetic divergence from the others.

In order to explain this prolonged coexistence within a single niche, we will examine mechanisms of phenotypic and genetic diversity within the simulation.

### Within niche phenotypic diversity

**FIGURE 3:**
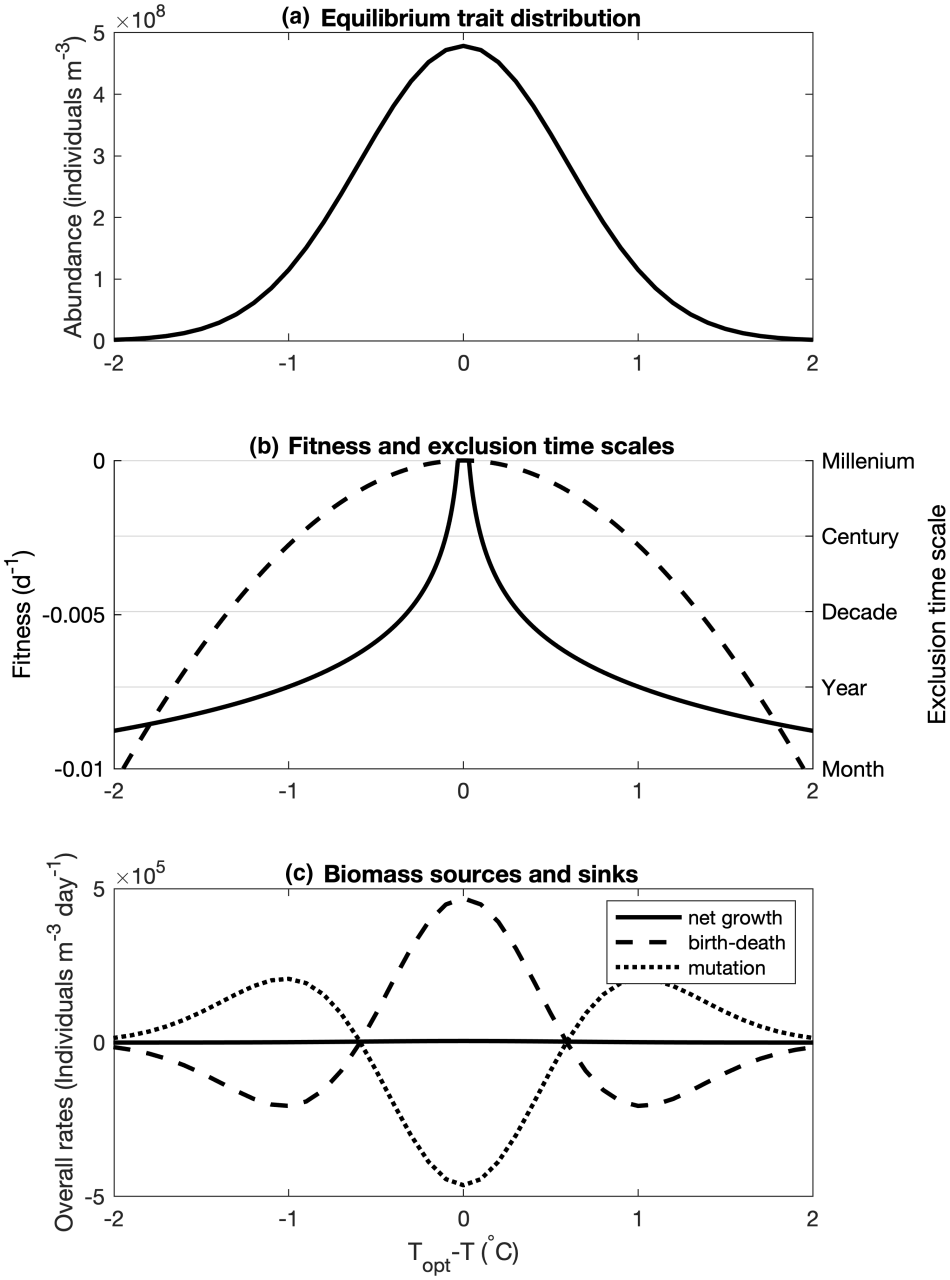
Trait distribution and mechanisms of coexistence. Panel (a) shows the eco‐evolutionary equilibrium distribution of phenotypes as a function of the thermal optimum minus the environmental temperature (*T*_*opt*_ ‐ *T*). Panel (b) shows the equilibrium net growth rate (or fitness landscape) in the absence of mutations (dashed line) and the associated time scales of competitive exclusion (solid line; calculated under the assumption that limiting nutrients are drawn down to the equilibrium requirement of the best‐adapted species ‐ see Appendix B). Time scales of competitive exclusion are calculated as the inverse of the net growth rate. Panel (c) shows the equilibrium balance of births‐deaths vs. mutation. Mutation acts as a sink for the best‐adapted phenotypes and as a source for maladapted phenotypes, thus supporting a broad distribution of traits with equal (zero) fitness.

Figure 3a shows the simulated distribution of traits at the end of the 1000‐year simulation. In a system without mutation, selection would drive the system towards dominance by a single optimally adapted phenotype. This can be seen in Figure 3b, in which the dashed line shows the expected net growth rates of different phenotypes at an ecological equilibrium (when nutrients are depleted to the minimum level required to support the best‐adapted phenotype; Tilman, 1980, see Appendix B). This fitness landscape shows that only the optimal phenotype can achieve a non‐negative net growth rate, and thus all other phenotypes should eventually go extinct. While the associated timescales of extinction (calculated as the inverse of the fitness landscape and shown by the solid line in Figure 3b) indicate that some phenotypes close to the optimum may take an extremely long time to go extinct, this is not sufficient to explain the trait distribution seen in Figure 3a – in a simulation of 1000 years duration, the timescales of exclusion suggest a much narrower distribution of surviving phenotypes.

Figure 3c shows that the equilibrium trait distribution is maintained by a mutation‐selection balance (Zhang & Hill, 2005), with imperfect heritability of traits serving to level out differences in net growth rate across the trait axis. A net excess of births over deaths around the optimal phenotype is exactly balanced by a mutational flux of individuals towards less favourable parts of the trait axis. This flux likewise supports a net excess of deaths relative to births further away from the optimal trait value.

Overall, the opposing forces of mutation and selection serve to flatten the fitness landscape (the solid line showing zero net growth in Figure 3c), which in principle would allow unlimited coexistence across the trait space. In practice, the breadth of the trait distribution is limited by the increasing likelihood of extinction for less well‐adapted (and hence less abundant) phenotypes. Nonetheless, the constant divergent flux of individuals provides a degree of standing trait variability.

### Within niche genotypic diversity

Is this mutational flattening of the fitness landscape sufficient to support the sustained divergence of genotypes seen in Figure 2? To explore this question, we modified the IBM to track the evolutionary trajectories of all simulated lineages, recording the time and phenotype (i.e. thermal optimum) associated with every cell division throughout the simulation.

**FIGURE 4:**
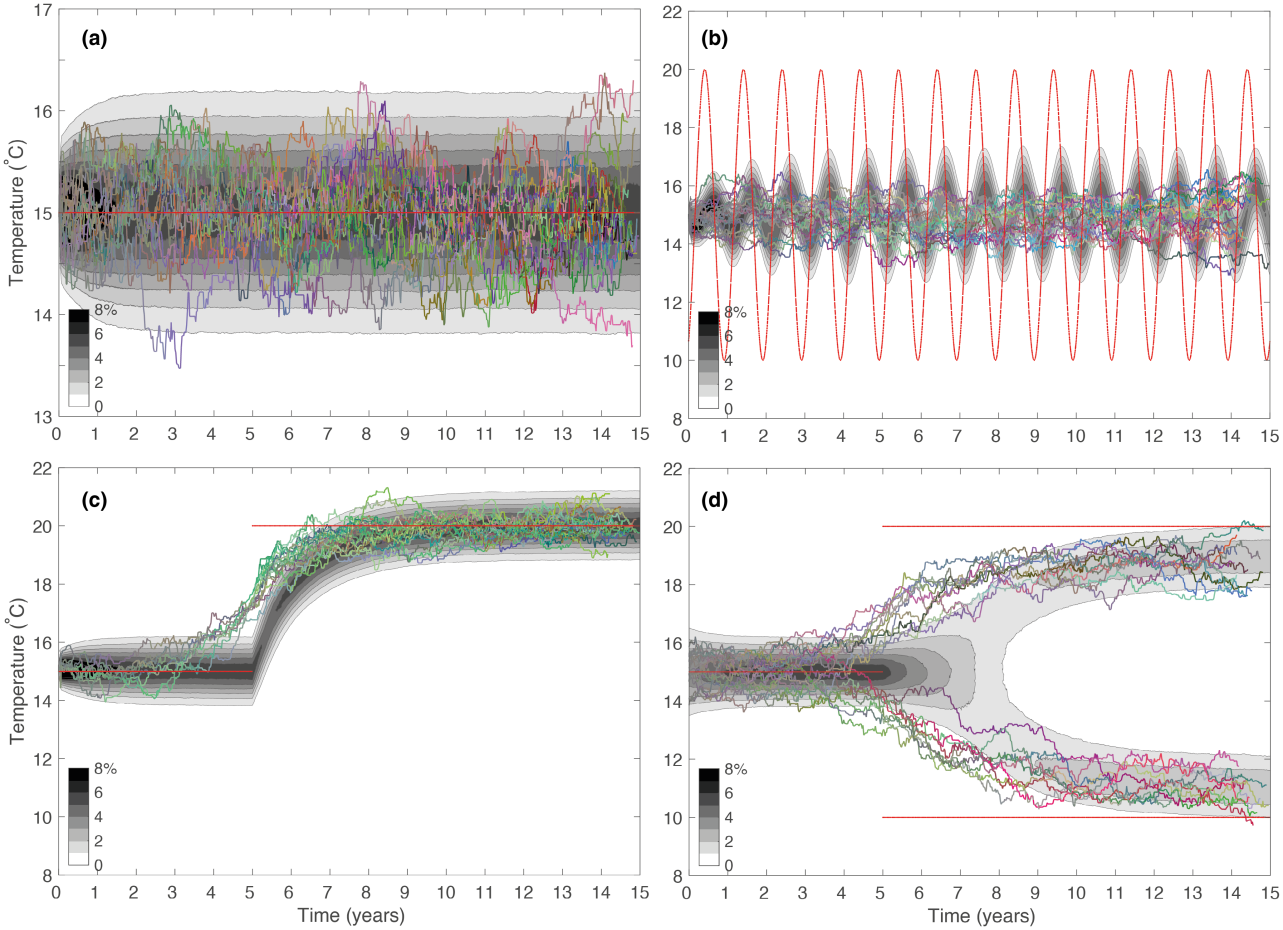
Eco‐evolutionary plankton dynamics during four 15‐year simulations with the IBM. Each simulation was seeded at *t* = 0 with a single cell with a thermal optimum of 15°C. Grayscale contours in each panel show the distribution of individuals among phenotypes through time. The branching lines show the genealogy of 20 cells sampled at random from cells alive during the final year of the simulation. Thermal phenotype is shown with the y coordinate, time of division with the x coordinate. Branch colours correspond to value of the neutral colour trait. The red lines show the range of environmental temperatures throughout each simulation: Panel (a) ‐ constant temperature; panel (b) ‐ sinusoidally varying temperature (mean 15°C, period 1 year, amplitude 10^°^C); panel (c) ‐ a constant temperature of 15°C increasing to 20°C at *t* = 5 years; panel (d) ‐ constant temperature until *t* = 5 years, and then changing between 10 and 20°C every 12 h (i.e. a squarewave with mean 15°C, period 1 day and amplitude 10°C).

This is shown in Figure 4a, which shows both the emergent abundance distribution during the first 15 years of the ‘constant temperature’ simulation described above and the evolutionary trajectories of 20 individuals that were sampled during the fifteenth year of that simulation. Each of these sampled cells can be tracked back through the generations to the initial seed, providing an exact genealogy with complete information regarding phenotypic changes at each generation.

The plotted trajectories in Figure 4a indicate that individual lineages, while centred around the optimal temperature, show considerable phenotypic variability throughout the simulation. This pattern again occurs through a balance of mutation and selection, as lineages move around the optimal trait value in a constrained random walk. Here, the introduction of trait variability is tempered at each generation as individuals with thermal optima further from the environmental temperature are less likely to successfully reproduce.

The simulated pattern of descent suggests two related characteristics. First, individual lineages are not associated with a single constant fitness on which selection can consistently act over long periods (even though the trait itself may be strongly and consistently correlated with fitness; Menden‐Deuer et al., 2021). Second, different lineages tend to exhibit similar average fitness over reasonably long time scales (decades or more). As a consequence, our simulations show extended coexistence of divergent lineages (Figure 2). While such a high degree of lineage divergence should be expected within a homogeneous population (Kingman, 1982), it occurs here for a group of competing and evolving lineages with clear differences in phenotype and associated fitness.

These results demonstrate that small random variations in individual fitness can give rise to identical average fitness, allowing populations to coexist indefinitely (Menden‐Deuer et al., 2021). In the following section, we will further test whether this mechanism is sufficiently robust to work in a temporally varying environment, under which adaptation to changing conditions might serve to accelerate competitive exclusion.

### Dynamic environmental forcing

Beckmann et al. (2019) explored the behaviour of the model in response to a number of alternative environmental forcing scenarios, with the simulated populations showing a clear adaptive response to each. We repeat those experiments here with identical model parameters (Table A.1), but over a slightly extended timescale of 15 years. Figure 4b,c shows the results of these simulations, which in all cases are ecologically consistent with the results presented by Beckmann et al. (2019).

**FIGURE 5:**
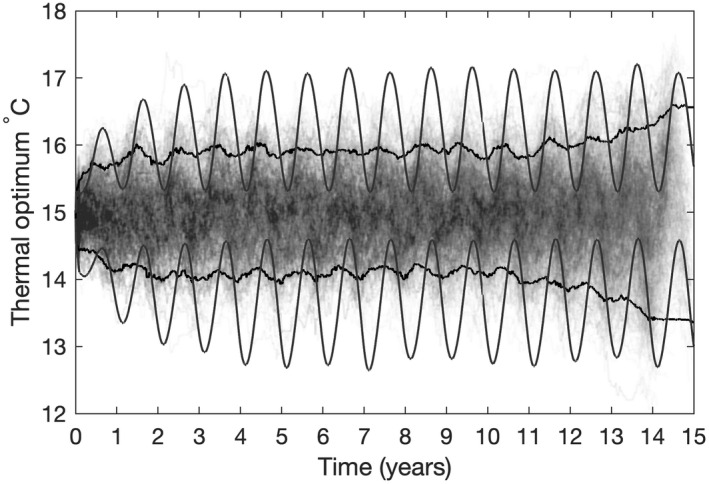
Evolutionary history of cells sampled in the last year of the simulation compared to abundance distributions throughout the simulation. The smooth black lines show the 5th to 95th percentiles of the abundance distribution at each point in the simulation. Evolutionary trajectories of 1000 cells sampled during the final year of a simulation are shown as grey lines. The 95th percentiles of this distribution are shown by the jagged black lines. Most of the cells sampled in the last year of the simulation (including those adapted to extremes of the temperature range) are descended from ancestors with thermal optima closer to the mean environmental temperature. Most of the cells that were adapted to extremes of temperature early in the simulation do not have descendants alive at the end of the simulation.

In Figure 4b, we introduced a sinusoidal annual cycle of ±5°C on top of the mean temperature of 15° (red lines). As was the case in a constant environment, the lineage tracking highlights a very high degree of lineage coexistence. Furthermore, while the 20 individuals sampled towards the end of the simulation are broadly distributed in terms of their thermal optima (between 13 and 17°C), they are descended from individuals with a narrower distribution of thermal optima early in the simulation. This is highlighted in Figure 5, which shows the 5th to 95th percentiles of the abundance distribution of all individuals throughout the simulation alongside the equivalent percentiles of the lineages sampled towards the end of the simulation. While the abundance distributions show that a significant number of individuals did adapt to the extremes of temperature, the lineage distributions show that very few of these survived to the end of the simulation. Adaptation to the extremes of temperature therefore appear to be an evolutionary dead‐end in this simulation, with phenotypes better adapted to the mean temperature most likely to survive in the long run.

In panels c and d of Figure 4, we explored the response of the system to an extreme change in the environmental forcing at *t* = 5 years. In panel c we instantly increased the average temperature by 5°C, while in panel d we added a strong daily temperature cycle (with the temperature changing instantly between 10 and 20°C every 12 h). The eco‐evolutionary responses to these changes again reflect the findings of Beckmann et al. (2019), with the simulated trait distribution either adapting to the warmer temperature (panel c) or branching into two distinct ecotypes adapted to the warmer and colder extremes of the fluctuating temperature range (panel d). In both cases, the plotted evolutionary trajectories reveal that the traits of sampled lineages all began changing towards the new optimal traits *before* the change in environmental conditions. While these changes increased the likelihood of extinction in the old environment, they provided a critical fitness advantage once the conditions changed.

In contrast to the classic pattern of a ‘hard’ selective sweep (Hermisson & Pennings, 2017; Maynard Smith & Haigh, 1974), through which a single beneficial mutation is fixed within a population, the patterns of evolution shown in Figure 4 are each characteristic of a ‘soft’ selective sweep (Hermisson & Pennings, 2017). When the environment changes (Figure 4b to d), standing phenotypic variation and rapid evolution allow multiple lineages to be carried through to the new environment, allowing greater coexistence within a single niche than might otherwise be predicted from the competitive exclusion principle.

### Hard vs. soft selective sweeps

**FIGURE 6:**
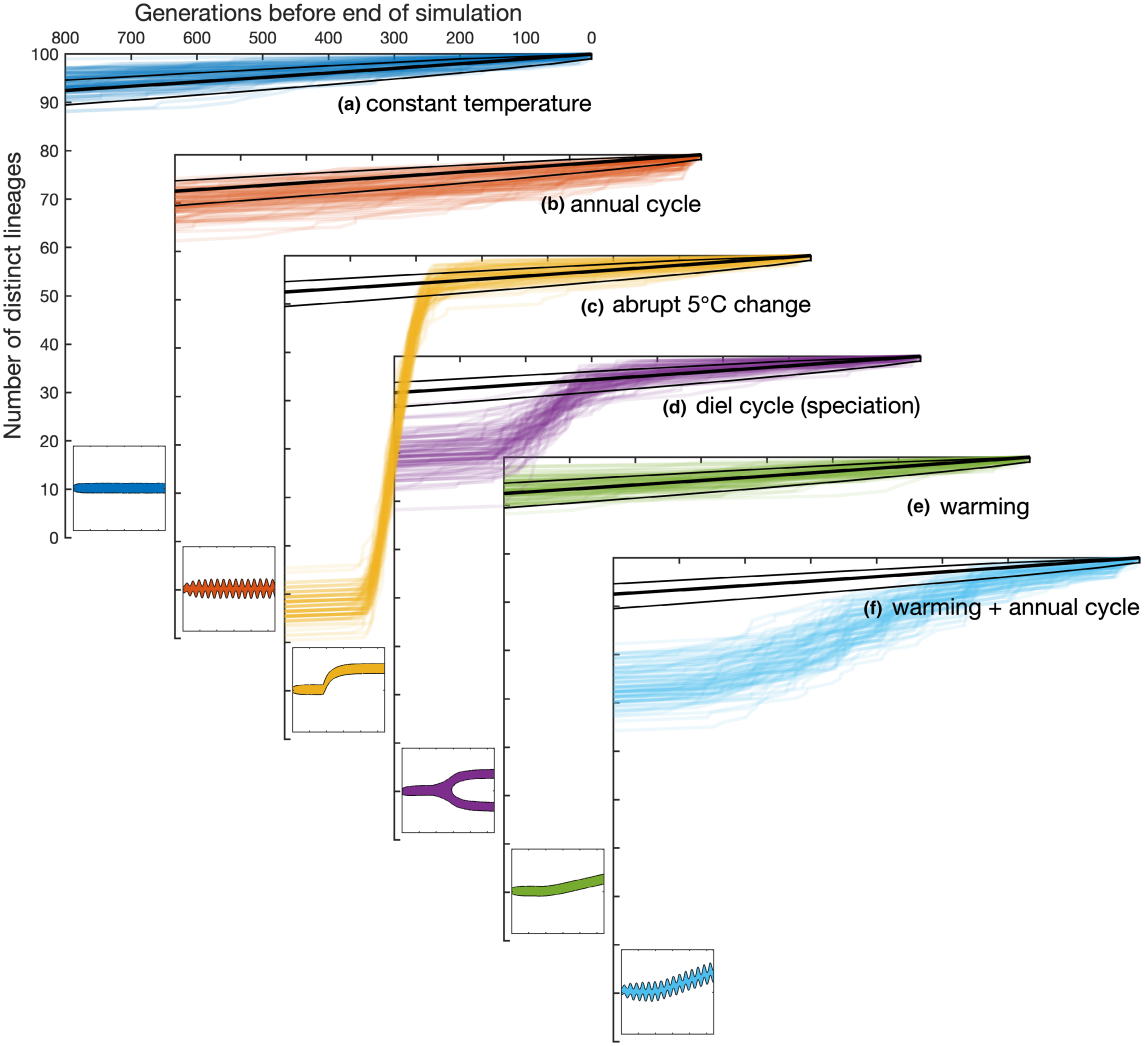
Patterns of coalescence under different environmental scenarios. Axes (a–d) correspond to the experiments shown in Figure 4. Axes (e–f) show results from two additional experiments: (e) 0.5°C per year warming applied from the end of year 5, (f) as for e, but with an annual cycle of ±5°C. in each case coalescence patterns are shown for 100 randomly selected phylogenies, in comparison the neutral model (black lines, mean ± 1 s.d.). inset panels show biomass as a function of time (x axis) and thermal optimum (y axis) for each experiment.

To more precisely quantify the degree to which evolution can ‘soften’ selective sweeps and maintain diversity in our simulations, we can compare each of the simulations above in terms of how many distinct lineages are carried through to the end of the simulation from earlier points in time. Among any 100 individuals randomly sampled at the end of a simulation, the expected number of unique ancestors they share must decrease as we move backwards through their evolutionary history. For a large and phenotypically homogeneous population in a constant environment, this pattern of coalescence is expected to occur very slowly by ecological drift, as indicated by the thick black lines in Figure 6 (see Appendix C and Halley & Iwasa, 2011).

In microbial populations under selection, coalescence is often predicted to happen much more rapidly, with a single beneficial mutation fixing very rapidly in a hard selective sweep (Louca et al., 2018). Panel a shows, however, that in a constant environment the simulated pattern of coalescence very closely follows the neutral model, with over 90% of the lineages remaining distinct through 800 generations. Furthermore, coalescence is only slightly accelerated with the introduction of a seasonal temperature cycle (Panel b). The sudden change in temperature of 5°C (Panel c) does introduce a relatively hard selective sweep, albeit with just under one third of the lineages successfully adapting to the change in conditions. The selective sweep is considerably less severe with the introduction of a daily temperature cycle (Panel d).

While these latter two experiments do lead to a significant loss of diversity, the introduced environmental perturbations are unrealistically severe, with temperature changing by 5–10°C in an instant. In panels (e) and (f), we introduce more realistic (although arguably still severe) changes, adding a 0.5°C per year warming (from the end of year 5) to the experiments with a constant temperature (e) and an annual cycle (f). In the absence of the seasonal cycle, this warming trend had almost no effect on the pattern of coalescence (panel e). However, when introduced to a simulation with a seasonal cycle, the warming trend led to a markedly more rapid loss of diversity. This likely occurs as yearly increases in temperature favour species adapted to the warmest part of the annual cycle over those adapted to the coldest temperatures.

## DISCUSSION

Perspectives on microbial life in the ocean are increasingly shaped by the vast amounts of molecular information made available by modern sequencing techniques (Mock et al., 2016). Despite a large and growing number of papers that provide realistic exceptions to the so‐called paradox of the plankton (Record et al., 2013), patterns of taxonomic diversity are regularly interpreted through a perspective of competitive exclusion and niche partitioning. A high degree of coexistence is often attributed to (potentially cryptic) niche separation (Louca et al., 2018)—but this strictly requires one hidden niche dimension for every additional coexisting species at equilibrium.

The neutral theory of biodiversity (Hubbell, 2001) provides an alternative view, attributing patterns of taxonomic diversity to the stochastic nature of births and deaths. Clusters of distinct individuals can emerge in the absence of any selective pressures, driven by the random process of ecological drift. Here, we show a similar result, but identify a clear mechanism by which neutrality can repeatedly emerge through rapid evolution driven by genetic, heritable epigenetic and heritable plastic changes.

This pattern of indefinite (albeit stochastic) coexistence can be understood from two perspectives. From a phenotypic perspective, the ecological components of the model point to dominance by a single ‘optimal’ phenotype under constant environmental conditions (Figure 3b). However, the mutational flux of individuals from better to worse adapted phenotypes effectively flattens the fitness landscape (Figure 3c), allowing unlimited coexistence. Alternatively, from a lineage‐based perspective, organisms do not have perfectly fixed traits from one generation to the next, and lineages thus occupy a distribution of traits around the optimal value. Over long periods, the average fitness of different lineages converge to effectively identical values, again allowing much longer periods of coexistence (set by population genetic rather than ecological considerations; Figure 6b).

These results are driven by a mutation‐selection balance that requires a dependable and relatively high rate of heritable trait changes. Following (Beckmann et al., 2019), we included heritable trait changes in the thermal optimum as Gaussian noise with a standard deviation of 0.1°C. While this may seem high, it is worth noting that thermal tolerance is affected by many genetic (Chakravarti et al., 2020) and otherwise heritable factors (McGuigan et al., 2021) and there are thus many potential pathways for this trait to evolve (Schaum et al., 2018). Thermal tolerance is also known to evolve extremely rapidly in response to environmental changes (~200 generations), even when such changes rely entirely on de novo variation and take place in asexual populations (Jin & Agustí, 2018; O'Donnell et al., 2018). Our simulated evolutionary trajectories (Beckmann et al., 2019) are not grossly out of alignment with responses observed in laboratory cultures (O'Donnell et al., 2018) or implied from field observations (Thomas et al., 2012). Further, running simulations with slower mutation rates prevented the model from showing any meaningful evolutionary response at all. Populations either remained unchanged (in response to sinusoidal forcing) or went extinct (in response to sudden temperature changes). Given the sheer size of microbial populations, and the ease with which they may generate the variation required to adapt extremely rapidly in laboratory experiments, the high rates of heritable trait change used in this model are reasonable.

It should however be noted that rarer and more stochastic trait changes might not lead to similar patterns of soft selective sweeps and extended coexistence. If a single large beneficial trait change occurs in isolation, it is likely to displace all other lineages over a timescale related to the associated increase in fitness. For example, we ran several simulations for which mutations occurred 10 times less frequently, but with a tenfold increase in variance. While this gave an identical rate of trait diffusion over many generations, the increased stochasticity of the simulation led to harder sweeps and rapid competitive exclusion in response to environmental change. Furthermore, evolution along a single trait axis (in this case thermal tolerance) presents a fairly large target for beneficial changes. It remains to be seen what patterns of coalescence will emerge in a model where evolutionary changes occur in multiple traits simultaneously. In a much larger multidimensional trait space, beneficial changes are likely to occur much less predictably, potentially shifting the system towards harder selective sweeps and stronger competitive exclusion.

These caveats notwithstanding, rapid evolution allows neutrality to emerge through a process of convergent and imperfect evolution and we see the sustained coexistence of phenotypically similar but genetically distinct lineages. This is a defining characteristic of functional redundancy (Louca et al., 2016; Louca et al., 2018). The assumptions of our model demonstrate that this phenomenon does not require the existence of additional hidden niche dimensions. Furthermore, our simulations suggest that high numbers of lineages are able to traverse even abrupt changes in environmental conditions (Figure 4), with the adaptive response to environmental changes underpinned by standing phenotypic variation and a steady stream of heritable trait changes, rather than the emergence of a single beneficial mutation. These patterns of evolution are characteristic of soft selective sweeps, which require either standing variation or a consistent supply of new beneficial mutations — both of which are extremely likely in highly abundant and rapidly reproducing microbial populations. Indeed, we were able to demonstrate the presence of soft sweeps in modelled populations on the order of only one million cells, somewhat less than the estimated 10^27^ *Prochlorococcus* cells currently alive in the ocean, or even the estimated effective population size of 10^13^ in a well‐mixed parcel of sea water (Kashtan et al., 2014).

Despite the inclusion of selection and environmental variability, our comparisons to the neutral model of coalescence suggest that hard selective sweeps are only likely to occur in the model under extremely rapid environmental changes that seem unlikely to occur over large spatial scales in a well‐mixed ocean. Several of our simulations remain consistent with a strictly neutral theory (Halley & Iwasa, 2011; Kingman, 1982), which predicts that the expected timescale of diversity loss will be proportional to the effective population size (Equation C.5). For the aforementioned well‐mixed population of *Prochlorococcus*, this is much longer than required to explain the observed (Kashtan et al., 2014) millions of years of divergence.

Our findings suggest that rapid evolution likely plays a key role in the coexistence of phenotypically similar but genetically distinct species in microbial communities, with functional redundancy emerging through convergent evolution. Nonetheless, our simulations remain highly idealized, in particular neglecting to account for dispersal and mixing of communities in a three‐dimensional environment. Further work is therefore required to explore the significance of soft selective sweeps in a metacommunity context.

## AUTHOR CONTRIBUTION

BAW designed the study and performed the research, BAW and SC wrote the manuscript.

### PEER REVIEW

The peer review history for this article is available at https://publons.com/publon/10.1111/ele.14061.

## Figures and Tables

**FIGURE A.1:**
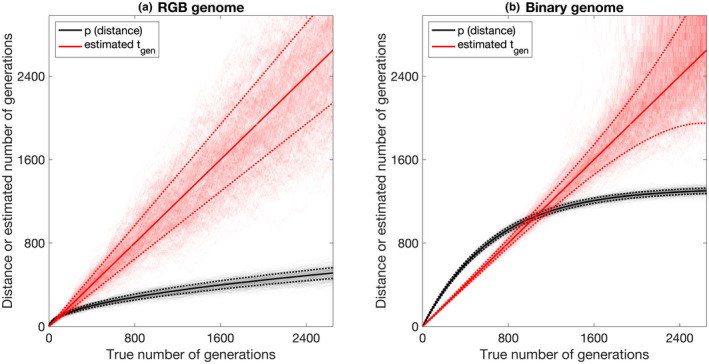
Theoretical and simulated accumulation of differences in the neutral genomes. Panel (a): RGB genome. The black lines shows the expected accumulation of Euclidean distances between the 50‐element rgb genomes (dashed lines are ±1 standard deviation). Panel (b): Binary genome. The black lines show the expected rate of accumulation of bitwise differences between 2350‐bit binary genomes (±1 standard deviation). In both cases, the thick red lines indicate the estimated number of generations relative to the true number of generations (±1 standard deviation). Pale lines show the simulated distribution of pairwise distances or estimated divergences among 25 individuals. While both genomes are encoded as 50‐element double precision vectors, it is clear that the binary genome gives a much more precise estimate of the number of generations, as long as the true number of generations is less than approximately half the number of bits.

**FIGURE A.2:**
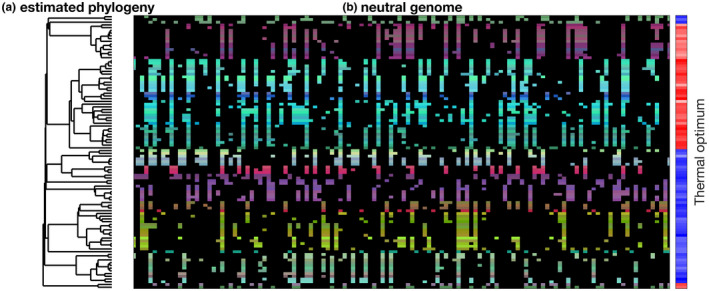
Phylogeny and neutral genome of 100 individuals sampled at the end of a simulation similar to the one shown in Figure 4d (but with a population of only ~5000). The dendrogram in panel (a) represents an estimated phylogeny derived from the binary genomes. Panel (b) shows the first 128 bits of the associated binary genomes. Zeros are black, while are ones shown with their neutral colour trait. The right‐hand colour scale shows the thermal optima of each individual (red = warm, blue = cold).

**FIGURE A.3:**
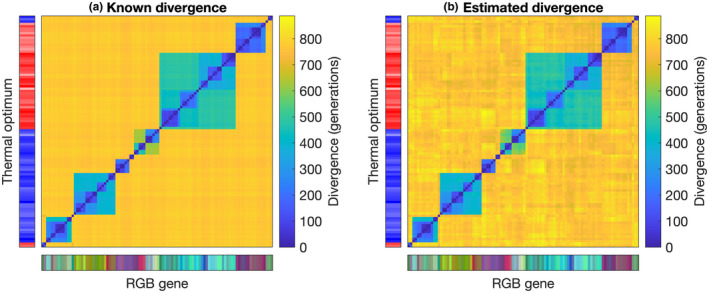
Heat maps showing pairwise distance matrices for the same 100 cells are presented in Figure A.2. Panel (a) shows known distances based on the lineage tracking. Panel (b) shows distances estimated from the neutral binary genomes. In each panel, the left‐hand colour scale shows the thermal optima of the sampled cells (red/blue = warm/cold adapted). The right‐hand colour scale shows the genealogical divergence in generations. The bottom colour scale shows the neutral colour traits of the sampled cells.

## Data Availability

Model code data have been deposited in GitHub (https://github.com/Ward‐Ecology/Plankton_IBM/tree/Plankton_IBM_1.4). The code release associated with the presented results has been assigned the DOI: 10.5281/zenodo.6583525.

## APPENDIX A Model description

### A.1 Individual‐based Eco‐Evo model

The eco‐evo model we develop builds upon the individual‐based model (IBM) presented by Beckmann et al. (2019). The model represents a closed system, in which individual phytoplankton (*b*_*i*_) grow as a function of temperature and nutrient availability, divide and die stochastically. Dead phytoplankton enter a detrital pool (*D*), which is converted back to inorganic nutrient (*N*) via a linear remineralisation term.

Cellular growth of individual phytoplankton (*μ*_*i*_) is defined in relation to a theoretical maximum of *μ*_0_ that is modified by temperature‐ and nutrient‐dependent functions (${ℱ}_{T}$ and ${ℱ}_{N}$).

(A.1)
$$\frac{{\mathrm{db}}_{i}}{\mathrm{dt}}=\mu_{i}=\mu_{0}\cdot b_{0}\cdot {ℱ}_{T}\cdot {ℱ}_{N}$$

Here, *μ*_0_ is the maximum doubling rate and *b*_0_ is the reference cellular biomass (Beckmann et al., 2019).

The thermal tolerance function decreases growth rate as the environmental temperature *T* deviates from a phytoplankton's thermal optimum (*T*_*opt*_). The breadth of the associated thermal niche is given by *θ*.

(A.2)
$${ℱ}_{T}\left(T\right)=\exp\left[-{\left(\frac{T-T_{\mathrm{opt}}}{\theta }\right)}^{2}\right]$$

Nutrient limitation is implemented with a Monod (1950) function, with a half‐saturation constant of *k*_*N*_.

(A.3)
$${ℱ}_{N}\left(N\right)=\frac{N}{k_{N}+N}$$

Individuals increase their cellular biomass at a rate set by their physiological traits (*μ*_0_, *T*_*opt*_, etc.) and the environmental conditions (*T* and *N*). Each cell grows until it reaches or surpasses a division threshold, which is set to twice its minimum viable biomass of *b*_0_. When this point is reached, the cell's biomass is divided equally between two daughter cells.

Mortality is applied stochastically, with cells having a fixed probability of death (*γ*_0_), at each time step. The number of live cells in the model thus changes according to the balance of individual divisions and deaths at each time step.

The overall phytoplankton biomass concentration in the model is calculated diagnostically as the sum of the biomass of all live cells.

(A.4)
$$P=\frac{1}{V}\sum_{i=1}^{M}b_{i}$$

where *M* is the number of live cells and *V* is the volume of the modelled culture. Note that we regulate the number of cells in the model by controlling the culture volume. We do not use the concept of super‐individuals°.

In contrast to the phytoplankton, which is treated as a collection of individuals, the nutrient and detrital pools are treated as homogeneous bulk variables. At each time step, uptake from the nutrient pool is taken as the sum of uptake by all individual cells, while production of detritus is taken as the combined biomass of all dying cells. Remineralisation from the detrital pool to the nutrient pool proceeds as a linear function of detrital biomass at each time step, with a mass‐specific rate of *τ*.

(A.5)
$$N_{t+1}=N+\left(\mathrm{\tau D}-\sum_{i=1}^{M}\mu_{i}\right)\Delta t$$

(A.6)
$$D_{t+1}=D+\left(\sum_{i=i_{\mathrm{die}}}b_{i}-\mathrm{\tau D}\right)\Delta t$$

where $i_{\mathrm{die}}$ is the index of all cells dying in a particular time step.

**Evolution**

Trait variation and inheritance are implemented in the IBM by assigning each newly divided cell the thermal optimum of its parent, perturbed by a value drawn from a Gaussian distribution with mean zero and standard deviation *σ*_*M*_.

$$T_{\mathrm{opt}}^{\prime }=T_{\mathrm{opt}}+\sigma_{M}$$

Changes in the thermal optimum affect the likelihood of survival by increasing or decreasing the individual's growth rate (Equation A.2), with better‐adapted individuals more likely to be reproduced in each subsequent generation.

### A.2 Phylogeny

#### A.2.1 Lineage tracking

At each cell division, we assign the two daughter cells a unique identity number. We also record the thermal optimum, time of division and the identity of the parent cell. This record is purged of extinct lineages at the end of each year in order to maintain the size of the associated files at a manageable level. This approach allows us to reconstruct evolutionary trajectories in the model with complete accuracy, as shown in Figure 4.

#### A.2.2 Ecologically neutral colour trait

While precise, the lineage tracking approach is also very expensive computationally and produces vast amounts of data. As an alternative approach, we added an ecologically neutral colour trait to identify closely related individuals.

The neutral colour trait is encoded as a heritable three‐element vector that corresponds to a unique colour in the red‐green‐blue (rgb) colour space.

$$\vec{\mathrm{rgb}}=\left[r,g,b\right]$$

The rgb vector is replicated at each cell division and each element then immediately undergoes a mutation, drawn from the standard normal distribution ($\phi \sim N\left[0,1\right]$).

$${\vec{\mathrm{rgb}}}^{\prime }=\vec{\mathrm{rgb}}+\vec{\phi }$$

As the value of the rgb vector has no effect on the fitness of the individual, changes in the rgb genome through generations are mathematically equivalent to a Gaussian random walk in a three‐dimensional space. The expected euclidean distance between two rgb vectors ${\hat{d}}_{\mathrm{rgb}}$ is therefore given as a function of the number of generations ($t_{\mathrm{gen}}$) since their most recent common ancestor

(A.7)
$${\hat{d}}_{\mathrm{rgb}}=\sqrt{4t_{\mathrm{gen}}}\cdot c$$

With a standard deviation of

(A.8)
$$\sigma_{{\hat{d}}_{\mathrm{rgb}}}=\sqrt{t_{\mathrm{gen}}}$$

Here, *c* is a correction factor that accounts for the number of dimensions, *n*_*rgb*_, using the ratio of two gamma functions.

$$c=\frac{\Gamma \left(\frac{n_{\mathrm{rgb}}+1}{2}\right)}{\Gamma \left(\frac{n_{\mathrm{rgb}}}{2}\right)}$$

While the distance between individuals in the rgb colour space can be used to estimate the time since their most recent common ancestor, the ratio of Equations A.8 and A.7 suggest an expected coefficient of variation (c.v.) of (2*c*)^−1^. For a three‐element rgb vector, the expected euclidean distance will be broadly distributed, with a standard deviation of ±44% of the expected value. Even if the rgb vector is extended to include 50 dimensions, the c.v. only drops to ±10%. This is somewhat imprecise (as shown in Figure A.1a), but the colour trait will be useful for identifying closely related individuals (e.g. Figure 1).

#### A.2.3 Binary genome

While the neutral colour trait is useful for visualization, it lacks the precision required to accurately track descent in the model. To achieve this we instead turn to the binary genome, for which each individual in the simulation is assigned a binary string of *L* = 2150 bits.

$$\vec{\mathrm{bin}}=\left[{\mathrm{bin}}_{1},{\mathrm{bin}}_{2},\ldots ,{\mathrm{bin}}_{L}\right]$$

In practice, the long binary string can be efficiently encoded as a 50‐element vector of floating point values, with 53 bits stored in the significand of each double‐precision element. (We could have stored 64 bits as unsigned integer values, but this was not computationally efficient given our code structure.)

At each generation, the binary genome is inherited from the parent cell and undergoes a single random bit‐flip with a probability of $p_{\mathrm{mut}}=1$. The bit to be flipped is drawn from a discrete uniform distribution; $R_{\mathrm{bin}}\sim U\left[1,L\right]$.

$${\mathrm{bin}}_{i}^{\prime }=\left\{\begin{array}{ll} 1-{\mathrm{bin}}_{i} & \text{if}\;R_{\mathrm{bin}}=i\\ {\mathrm{bin}}_{i} & \text{else} \end{array}\right.$$

With one randomly selected bit flipped at an average rate of once every 1∕*p*_*mut*_ generations, the expected normalized Hamming distance between two binary genomes (${\hat{d}}_{\mathrm{bin}}$) is given as a function of the number of generations ($t_{\mathrm{gen}}$) since their most recent common ancestor.

(A.9)
$${\hat{d}}_{\mathrm{bin}}=\frac{1}{2}\left[1-\exp^{\left(-\frac{4\cdot t_{\mathrm{gen}}\cdot p_{\mathrm{mut}}}{L}\right)}\right]$$

with a standard deviation of

(A.10)
$$\sigma_{{\hat{d}}_{\mathrm{bin}}}=\sqrt{\frac{{\hat{d}}_{\mathrm{bin}}\left(1-{\hat{d}}_{\mathrm{bin}}\right)}{L}}$$

These two equations (visualized in Figure A.1b) show that ${\hat{d}}_{\mathrm{bin}}$ increases predictably with the number of generations, saturating at 0.5 as the number of mutations approaches the length of the binary genome (*L*). The non‐linearity of the apparent trend is attributable to unobservable multiple flips of the same bits (homoplasy), as predicted by the two‐base Jukes and Cantor model (black line).

It is also clear that ${\hat{d}}_{\mathrm{bin}}$ increases in a much more predictable way than ${\hat{d}}_{\mathrm{rgb}}$ (as long as the number of mutations remains less than approximately half the number of bits in the binary genome). This makes it a much better candidate for use as a molecular clock.

Accordingly, the estimated number of generations, ${\hat{t}}_{\mathrm{gen}}$ since the divergence of any two lineages can be estimated from the simulated Hamming distance, *d*, between their binary genomes.

(A.11)
$${\hat{t}}_{\mathrm{gen}}=-\frac{1}{4p_{\mathrm{mut}}}\ln\left(1-2d\right)$$

with standard deviation

(A.12)
$$\sigma_{{\hat{t}}_{\mathrm{gen}}}=\frac{1}{p_{\mathrm{mut}}}\sqrt{\frac{d\left(1-d\right)}{4L{\left(1-2d\right)}^{2}}}$$

Equation A.12 and Figure A.1b demonstrate that the binary genome can be used to estimate divergence with a high degree of precision, as long as *t*_*gen*_ < *L*∕2 (N.B. longer simulations can be resolved by decreasing the probability ($p_{\mathrm{mut}}$) of a bit flip at each generation)

The demonstrated precision of the binary clock (Figure A.1) allows us to reconstruct the simulated phylogeny without the expense of recording every single division event. The basic principles of the binary clock are shown in Figure A.2. The dendrogram in Panel a shows the estimated phylogenetic tree for 100 individuals sampled from a simulation similar to the one shown in Figure 4d (but with a much smaller population size of ~5000 to allow a more structured tree). Panel b shows the first 128‐bits of the corresponding binary genomes (one row for each of individual). The known distance matrix from the lineage tracking is compared to the equivalent distance matrix estimated from the binary genome in Figure A.3.

**TABLE A.1:** Standard model parameters

Model parameter	Symbol	Value	Units
Total nutrient load	*N*_*t*_	5	mmol N m^−3^
Maximum cellular growth rate	*μ*_0_	ln2	d^−1^
Minimum cellular biomass	*b*_0_	5 × 10^−10^	mmol N
Nutrient half‐saturation	*k*_*N*_	0.15	mmol N m^−3^
Linear mortality rate	*γ*_0_	0.1	d^−1^
Remineralisation rate	*Τ*	0.25	d^−1^
Thermal optimum	*T*_*opt*_	Variable	°C
Breadth of thermal niche	*Θ*	6	°C
Standard deviation of ‘mutations’	*σ*_*M*_	0.1	°C
Time step	Δ*t*	1/24	d
Volume of growth culture	*V*	10^−4^	m^3^

## APPENDIX B EQUILIBRIUM SOLUTIONS IN FIGURE 3

### B.1

Figure 3 was created using the ‘trait‐diffusion’ version of the model, as described by Beckmann et al. (2019). In this version of the model the community is represented by the 201‐element vector, $\vec{P}$, corresponding to 201 different thermal optima. The equation for phytoplankton growth is then

(B.1)
$$\frac{d\vec{P}}{\mathrm{dt}}=\vec{\mu }\cdot \vec{P}-T\vec{P}-\gamma_{0}\vec{P}$$

Here, $\vec{\mu }$ is the nutrient‐ and temperature‐limited gross growth rate, T is a trait‐diffusion matrix that describes the fraction of daughter cells diverted to neighbouring phenotypic compartments at each generation, and *γ*_0_ is the linear mortality rate.

Figure 3a shows the equilibrium biomass distribution of $\vec{P}$ in a constant environment, plotted as a function of the thermal optimum minus the environmental temperature (*T*_*opt*_ ‐ *T*). This is identical to the trait distribution produced by the individual‐based model used in the rest of the manuscript. The equilibrium birth‐deaths in Figure 3c are given by $\vec{\mu }\cdot \vec{P}-\gamma_{0}\vec{P}$. The equilibrium mutation rates are given by $T\vec{P}$

In contrast to the results shown in Figure 3a and c, Figure 3b shows the equilibrium fitness landscape around the optimally adapted population *P*_*opt*_ in a system without mutation (i.e. T is replaced by a zero matrix). This system reaches an equilibrium when the growth rate of the best‐adapted species (*T* = *T*_*opt*_) is exactly matched by the mortality rate.

(B.2)
$$\mu_{0}\cdot {ℱ}_{N}\left(N\right)\cdot {ℱ}_{T}\left(T\right)=\gamma_{0}$$

Given that ${ℱ}_{T}\left(T\right)=1$ when *T* = *T*_*opt*_, at equilibrium the following is true for the optimally adapted population

(B.3)
$$\mu_{0}\cdot {ℱ}_{N}\left(N\right)=\gamma_{0}$$

As all phenotypes have identical nutrient traits, the net growth rate (or fitness) of each phenotype can then be calculated as

(B.4)
$$\mu_{\mathrm{net}}=\gamma_{0}\cdot {ℱ}_{T}\left(T\right)-\gamma_{0}$$

This is zero for the optimally adapted phenotype, and less than zero for all others. The exclusion time scale is simply the negative of the inverse of this value.

## APPENDIX C NEUTRAL MODEL OF LINEAGE COALESCENCE

### C.1

Simulated rates of coalescence through time in Figure 6 are compared to predictions of a neutral theory model (Halley & Iwasa, 2011; Kingman, 1982). Going backwards in time, for a population of *N* individuals the per generation probability of a single coalescence event among *k* lineages is given by

(C.1)
$$p=\frac{k\left(k-1\right)}{2N}$$

This gives an expected waiting time for coalescence *T* (in generations) of

(C.2)
$$\mu_{T}=p^{-1}$$

with a standard deviation of

(C.3)
$$\sigma_{T}=\sqrt{\frac{1-p}{p^{2}}}$$

The expected number of lineages can also be expressed as a function of time *t* (in generations),

(C.4)
$$k\left(t\right)=\frac{k_{0}}{1+\frac{t}{t_{\text{half}}}}$$

where *k*_0_ is the number of sampled lineages at *t* = 0 and $t_{\text{half}}$ is given by

(C.5)
$$t_{\text{half}}=\frac{2N}{k_{0}}$$
